# Ciprofloxacin Exerts Anti-Tumor Effects In Vivo Through cGAS-STING Activation and Modulates Tumor Microenvironment

**DOI:** 10.3390/cells14131010

**Published:** 2025-07-02

**Authors:** Jian-Syun Chen, Chih-Wen Chi, Cheng-Ta Lai, Shu-Hua Wu, Hui-Ru Shieh, Jiin-Cherng Yen, Yu-Jen Chen

**Affiliations:** 1 Institute of Pharmacology, College of Medicine, National Yang Ming Chiao Tung University, Taipei 112304, Taiwan; manchette.4320@mmh.org.tw; 2 Division of Colon and Rectal Surgery, Department of Surgery, MacKay Memorial Hospital, Taipei 104217, Taiwan; iamctlai.4987@mmh.org.tw; 3 Department of Medicine, MacKay Medical College, New Taipei City 252005, Taiwan; 4 Department of Medical Research, MacKay Memorial Hospital, New Taipei City 251020, Taiwan; b514.b514@mmh.org.tw (C.-W.C.); sophia1000522.b750@mmh.org.tw (S.-H.W.); ru123@mmh.org.tw (H.-R.S.); 5 Department of Radiation Oncology, MacKay Memorial Hospital, Taipei 104217, Taiwan; 6 Department of Artificial Intelligence and Medical Application, MacKay Junior College of Medicine, Nursing and Management, Taipei 112021, Taiwan; 7 Department of Medical Research, China Medical University Hospital, Taichung 404332, Taiwan

**Keywords:** ciprofloxacin, cGAS, STING, tumor microenvironment

## Abstract

Immunotherapy targeting the immune functions of the tumor microenvironment (TME) is beneficial for colorectal cancer; however, the response rate is poor. Ciprofloxacin is a fluoroquinolone-class antibiotic that is used to treat bacterial infections. The purpose of this study is to assess the mechanism of ciprofloxacin that enhances anti-PD1 in colorectal cancer. We found that ciprofloxacin induced cytosolic DNA, including single-stranded and double-stranded DNA, formation in mouse CT26 colorectal adenocarcinoma cells. Molecules in DNA-sensing signaling such as cGAS, STING, and IFNβ mRNA and protein expression were elicited after ciprofloxacin treatment in CT26 cells. STING siRNA abrogated the cGAS-STING pathway activation by ciprofloxacin. In vivo, ciprofloxacin exhibited a synergistic effect with anti-PD1 to suppress tumor growth in a CT26 syngeneic animal model without biological toxicity. The examination of TME revealed that ciprofloxacin, alone and in combination therapy, induced M1 and red pulp macrophage production in the spleen. In tumors, M1 and M2 macrophage levels were increased by ciprofloxacin, and CD8^+^ T cell granzyme B expression was increased after combination therapy. STING showed the highest expression in tumor specimens after combination treatment. Ciprofloxacin may enhance the anti-PD1 efficacy and modulate the TME through the cGAS-STING pathway.

## 1. Introduction

Global statistics show that colorectal cancer (CRC) is the third most common cancer and is ranked the second most common cause of cancer-related death [1]. CRC treatments include radical surgery, chemotherapy, radiation therapy, concurrent chemoradiation therapy, and targeted therapy [2]. In clinical settings, about 20% of patients with CRC are identified as advanced stage, and among them, 40% of patients with CRC display recurrence after the treatment of localized tumors. The prognosis of recurrent or metastatic CRC is not satisfactory, and the five-year survival rate is less than 20% [3]. The development of new strategies for CRC treatment remains an ongoing issue.

Immunotherapy has brought cancer therapy into a new era, and it has been approved for several cancer types, including CRC [4]. Two negative regulatory molecules, cytotoxic T lymphocyte-associated protein 4 and programmed cell death protein 1 (PD-1), are expressed on T cells that downregulate the immune response and prevent excessive inflammation. Immune checkpoint inhibitors block negative regulatory signals and restore T cell immune function to restore the anti-tumor effect. Despite the success of different cancer treatments, the response rate to immune checkpoint inhibitors is quite low, ranging from 20% to 40% [5]. Accumulating evidence suggests that the tumor response to immune checkpoint inhibitors is highly dependent on tumor immunogenicity [6]. The combination of immunogenic cell death-inducing drugs with immunotherapy may enhance anti-tumor efficacy and expand the benefits of immunotherapy.

The tumor microenvironment (TME) is a complex ecosystem that promotes tumor initiation, progression, invasion, and metastasis [7]. It contains cancerous and non-cancerous cells, including various immune cell types, cancer-associated fibroblasts, surrounding blood vessels, extracellular matrices, vascular endothelial cells, and pericytes. The reciprocal interaction of tumor and non-tumor cells remodels the environment to support nutrients and provide an excellent space for tumor growth [7]. Immune cells in the TME play different roles in exerting anti-tumor effects or escaping immunosurveillance effects through innate or adaptive immune responses. Several immune cell types, such as natural killer (NK) cells, dendritic cells (DCs), myeloid-derived suppressor cells, and CD8^+^ cytotoxic T cells, function as tumor suppressors by secreting cytokines or chemokines. Another subpopulation of T cells is regulatory T cells (Tregs), which play a suppressive role in immune response and promoting tumor development. Macrophages are classified into two subtypes: those that polarize to M1 macrophages that exhibit anti-tumor activity and those that polarize to M2 macrophages that promote tumor growth [8,9].

The intracellular DNA-sensing signal cGAS-STING pathway is a defense system that is activated by the detection of foreign pathogens or bacterial invasion to mediate innate immune functions [10]. DNA released from the nucleus damaged by chemotherapeutic agents or radiotherapy or self-DNA released from tumor cells is transferred into the cytosol and the formation of micronuclei occurs [11]. DNA is exposed in the cytosol for cGAS surveillance when the nuclear envelope of the micronuclei ruptures. When cytosolic DNA containing double-stranded DNA (dsDNA) and single-stranded DNA (ssDNA) is exposed, it binds to cGAS and is converted into the secondary messenger cyclic GMP-AMP (cGAMP). cGAMP binds to STING located in the endoplasmic reticulum and is translocated to the Golgi apparatus [12,13]. This subsequently recruits and activates TANK-binding kinase 1 (TBK1) and transcription factor IFN regulatory factor 3 (IRF3) and induces the production of type I interferon (IFN) or other cytokines [14,15]. This DNA-sensing signaling pathway contributes to tumorigenesis and metastasis by evading immune cell surveillance. Therefore, targeting the cGAS-STING pathway to enhance cancer immunotherapy may be an option for cancer treatment [16]. However, their therapeutic efficacy in patients with colon cancer remains limited.

Ciprofloxacin (ciproxin) is a broad-spectrum antibiotic belonging to the fluoroquinolone class. Ciprofloxacin exerts antibacterial effects in the treatment of the urinary tract, the respiratory tract, the gastrointestinal tract, pneumonia, skin and soft tissue, sepsis, bone and joint infections, and sexually transmitted infections, and was approved by the U.S. Food and Drug Administration in 1987 [17]. The mechanisms of action of ciprofloxacin entail the inhibition of the activity of DNA gyrase (topoisomerase II) and interference in bacterial DNA replication and repair processes, ultimately leading to bacterial death [18]. The topoisomerase II inhibitor teniposide mediates the cGAS-STING axis and induces tumor immunogenicity by inducing the release of high-mobility group box 1 (HMGB1) and type I IFN signaling in tumor cells. Teniposide-treated tumor cells can activate the anti-tumor T cell response and DC maturation both in vitro and in vivo, and potentiate the anti-tumor efficacy of anti-PD1 in mouse multiple-type tumor models [19].

However, whether ciprofloxacin mediates the cGAS-STING signaling pathway to suppress tumor growth remains unknown. Therefore, in this study, we focus on the effect of ciprofloxacin anti-tumor activity and analyze its potential therapeutic effect in augmenting anti-PD1 activity against colon cancer.

## 2. Materials and Methods

### 2.1. Colorectal CT26 Cell Culture

Murine colon adenocarcinoma CT26 cells, purchased from the American Type Culture Collection (Manassas, VA, USA), were maintained in a culture medium containing the Roswell Park Memorial Institute (RPMI)-1640 medium (GIBCO, Grand Island, NY, USA) replenished with 10% fetal bovine serum (FBS) (Hyclone, Logan, UT, USA) and 2 mM L-glutamine (Merck, Darmstadt, Germany). CT26 cells were grown and maintained under specific conditions, i.e., at a temperature of 37 °C and in a humidified 5% CO_2_ incubator. The cells were maintained in a growth medium till the cells in their log phase reached 80% confluence; then, they were sub-cultured three times per week.

### 2.2. Cell Viability Assay

The 3-(4, 5-dimethylthiazol-2-yl)-2, 5-diphenyltetrazolium bromide (MTT) assay was used to detect the intracellular mitochondrial enzyme activity of cells, in which a formazan product was formed that indicated cell viability. CT26 cells were cultured in 24-well plates and incubated with different concentrations of ciprofloxacin for 24, 48, and 72 h. After this incubation with ciprofloxacin, cells were washed with phosphate-buffered saline (PBS) and placed in the MTT solution for 4 h at 37 °C. The solution was aspirated, and the formazan crystals deposited inside the cells were dissolved using dimethyl sulfoxide for 30 min. The absorbance of the samples was measured at 570/630 nm using an ELISA reader (Molecular Devices, San Jose, CA, USA).

### 2.3. Morphology Observation

Cells were grown in 6-well culture plates overnight and followed by incubation with 4 and 16 μM of ciprofloxacin for 24, 48, and 72 h. The cells were washed with PBS and Liu’s A solution was added for 45 s, then Liu’s B solution (Muto Pure Chemicals Co., Ltd., Bunkyou-Ku, Tokyo, Japan) was added for 90 s to stain the cells. The BX51 light microscope (Olympus, Tokyo, Japan) was applied to visualize the cell morphology, and the cell morphology was photographed using a CCD camera (SPOT Imaging, Sterling Heights, MI, USA).

### 2.4. Cell Cycle Analysis

CT26 cells were treated with ciprofloxacin for 24, 48, and 72 h. After treatment, cells were harvested and washed with PBS, and 70% ethanol was used to fix the cells for 10 min. The fixed cells were then washed with cold PBS, 10 mg/mL RNase was used to lyse the RNA, and the cells were stained with 1 mg/mL propidium iodide at 37 °C for 30 min in the dark. The DNA histogram of the cells was analyzed using flow cytometry (BD FACSCalibur, Becton Dickinson, Lincoln Park, NJ, USA). The percentages of G0/G1, S, and G2/M phase acquisition in 10^4^ cells were analyzed using the software ModFit LT (version 4.0). The cell cycle experiments were performed in triplicate.

### 2.5. Detection of Cytosolic DNA Concentration

Cells treated with ciprofloxacin were washed with PBS, trypsin was used to detach the cells, and the cells were centrifuged at 1200 rpm for 5 min at 4 °C to assemble them. Commercial lysis buffer (Chemicon^®^ Cytoplasmic Lysis Buffer, Merck Millipore, Burlington, MA, USA) was used to solubilize and break up the cells. The disrupted cells were then centrifuged at 1200 rpm for 5 min at 4 °C, and the supernatant containing cytosolic DNA (including mitochondrial DNA) was collected. The spectrophotometer (NanoDrop2000C, Thermo Fisher Scientific Inc., Waltham, MA, USA) was used to detect total and single-stranded DNA contents based on the absorbance at the wavelength of 260 nm. The concentration of dsDNA was determined by subtracting the quantity of ssDNA from that of the total DNA.

### 2.6. mRNA Expression Analysis

After ciprofloxacin treatment, the medium was removed, and RNAzol^®^ RT reagent (Molecular Research Center, Inc., Cincinnati, OH, USA) was added for 15 min to disrupt cells, and total RNA was isolated from the samples. The RNA concentration was determined by measuring the absorbance at 260 nm using the NanoDrop2000C Spectrophotometer. Subsequently, 1 µg of RNA was used to synthesize cDNA using a RevertAid First Strand cDNA Synthesis Kit (Thermo Fisher Scientific, Waltham, MA, USA). The cDNA was then used as the template to perform quantitative reverse transcription PCR (qPCR) using a LightCycler^®^ 96 real-time PCR device (Roche, Penzberg, Upper Bavaria, Germany). qPCR programs were performed as follows: at 95 °C for 3 min for hot start; 40 cycles at 95 °C for 3 s, 65 °C for 30 s, and 72 °C for 3 min, and finally held at 4 °C. Primers used for the cGAS-STING pathway genes were as follows: cGAS, forward 5′-TGAACATGTGAAGATTTCTGCTCC-3′ and reverse 5′-TGACTCAGCGGATTTCCTCG-3′; STING, forward 5’-ACTGCCGCCTCATTGTCTAC-3′ and reverse 5′-ATGGGGGCATTCATGGTA-3′; IRF3, forward 5′-CGGAGGCTTAGCTGACAAAGA-3′ and reverse 5′-ATGCTCTAGCCAGGGGAGGA-3′; IFNβ, forward 5′-CGGAGGCTTAGCTGACAAAGA-3′ and reverse 5′-ATGCTCTAGCCAGGGGAGGA-3′; and actin, forward 5′-GCCAACCGTGAAAAGATGAC-3′ and reverse 5′-GAGGCATACAGGGACAGCAC-3′. The actin expression level was used as an internal control to normalize the expression of other mRNAs in the samples.

### 2.7. Western Blot

After treatment, cells were lysed with a lysis buffer for 30 min at 4 °C (Cell Signaling Technology, Danvers, MA, USA) to harvest whole-cell lysate and then centrifuged at 15,000× *g* for 15 min at 4 °C. The supernatants were collected, and the bicinchoninic acid protein assay kit (Bio-Rad Laboratories, Hercules, CA, USA) was used to determine the protein concentrations. The lysates were boiled for 10 min at 100 °C to denature proteins, and an equal mass of denatured proteins was loaded onto sodium dodecyl sulfate–polyacrylamide gels for running, and subsequently, the proteins were transferred to polyvinylidene difluoride (PVDF) membranes. The PVDF membranes were soaked with the blocking buffer (containing 5% defatted milk) at room temperature for 1 h with gentle agitation to prevent non-specific binding. Subsequently, the membranes were incubated with primary antibodies against the detected proteins cGAS (GeneTex, Irvine, CA, USA), STING (Cell Signaling Technology, Danvers, MA, USA), IFNβ (Abcam, Cambridge, UK), and actin (GeneTex) at 4 °C overnight. Then, PVDF membranes were incubated with a secondary antibody solution (Jackson ImmunoResearch, West Grove, PA, USA) conjugated with horseradish peroxidase for 1 h at room temperature. The membranes were applied with the enhanced chemiluminescence substrate (GeneTex), and signals were captured using a CCD camera-based image machine, i.e., the Multigel-21 Multi-Function Gel Image System (Top Bio Co., Ltd., Lin Kou, New Taipei City, Taiwan). The band intensity was analyzed using the software ImageJ Version 1.54i, and the internal control level was normalized. β-actin was used as the internal control.

### 2.8. Syngeneic Tumor Animal Model

Five-week-old male BALB/c mice were obtained from the National Laboratory Animal Center of Taiwan (Taipei, Taiwan) and kept under a standard 12 h/12 h light/dark cycle in a specific pathogen-free facility. The experimental protocols followed the rules and regulations of the Affidavit of Approval of Animal Use Protocol [20], and were approved by the Institutional Animal Care and Use Committee (IACUC) of MacKay Memorial Hospital (IACUC number: MMH-A-S-106-01). Subsequently, 4 × 10^6^ CT26 colon cells in saline were hypodermically implanted into the right hind limbs of the mice, and CT26 colon tumors were allowed to grow to a diameter of approximately 0.5 cm. The mice were randomly assigned to four treatment groups: Group I, control; Group II, anti-mouse PD-1 antibody (200 μg administered through an intraperitoneal injection every other day for a total of three times per mouse) clone RMP1-14 obtained from Bio X cell, Lebanon, NH, USA; Group III, ciprofloxacin (30 mg/kg/d administered through oral gavage for 10 consecutive days); and Group IV, a combination of ciprofloxacin and anti-PD1.

### 2.9. Isolation of Macrophages from Mouse Spleen

BALB/c mice were euthanized, and their spleen was isolated, ground, and filtered through a 70 μm cell strainer. The specimens were added to HISTOPAQUE 1083 (Sigma-Aldrich, St. Louis, MO, USA) and centrifuged at 2000 rpm for 20 min at room temperature. The mononuclear cell layer was isolated and collected in Hank’s balanced salt solution (HBSS). The cells were washed with HBSS and centrifuged at 1200 rpm for 10 min twice. The cells were cultured in the RPMI-1640 medium containing 10% FBS and 1% penicillin/streptomycin antibiotics in an incubator with 5% CO_2_ at 37 °C. After 7 d, the adherent cells were stimulated with lipopolysaccharide (100 ng/mL, Sigma-Aldrich) and IFN-γ (20 ng/mL, Bio-Techne, Minneapolis, MN, USA) to allow them to polarize to M1 macrophages.

### 2.10. Measurement of Tumor Size and Evaluation of Biochemical Toxicity

The tumor size and body weight of each mouse were recorded and calculated every other day. Tumor size was measured with an electronic caliper and calculated using the following formula: 0.5 × L × W^2^, where L denotes the length and W denotes the width of the tumor. White blood cell count and liver and kidney function were used as indicators of biochemical toxicity after the administration of ciprofloxacin or anti-PD1 antibodies. Blood samples were gathered from the retro-orbital sinus every week. The number of white blood cells in the blood was counted using an automatic Coulter counter (HEMAVET HV950; Drew Scientific Inc., Plantation, FL, USA). The plasma levels of alanine transferase and creatinine in the blood, representing liver and kidney function, were determined with plasma samples loaded on Fuji Dri Chem slides (FUJIFILM Corporation Asaka Technology Development Center, Minamiashigara-shi, Kanagawa, Japan) and detected using an analyzer machine (Fujifilm DryChem NX-500, FUJIFILM Corporation, Tokyo, Japan).

### 2.11. Flow Cytometry to Detect Immune Cell Expression in Tumor and Spleen

At the end of treatment, animals were anesthetized with ketamine (100 mg/kg) and xylazine (10 mg/kg) and euthanatized. The tumor samples were removed, chopped into small species, and digested with DNase I (10 μg/mL) (Sigma-Aldrich) and Liberase TM Research Grade (25 μg/mL) (Roche, Basel, Switzerland) solution for 30 min at 37 °C. The suspension was sieved using a 70 μm cell strainer and then centrifuged at 1200 rpm for 5 min at 4 °C to collect single cells. Spleen tissues were harvested, ground, and filtered using a 120 μm cell strainer. To obtain a single-cell suspension, the samples were centrifuged at 1800 rpm for 5 min at 4 °C. The red blood cells of all samples were lysed using the ammonium–chloride–potassium lysis buffer (Invitrogen, Waltham, MA, USA). Subsequently, cells were incubated with rat IgG (1 μg/1 × 10^6^ cells, Sigma-Aldrich) for 15 min at 37 °C to diminish the non-specific binding before dyeing the immune cells with specific surface markers. Fluorochrome-conjugated mouse antibodies were added and incubated for 30 min at 4 °C. The antibodies conjugated with fluorochromes included the following: anti-CD45-Brilliant Violet 510TM, anti-PD1-APC/Cyanine7, anti-PD-L1-PE/DazzleTM 594, anti-CD3-FITC, anti-CD11b-Brilliant Violet 605TM, anti-Ly6G-PE/Cyanine7, anti-NKG2D-PE, anti-MHCII-Brilliant Violet 650TM, anti-CD11b-Brilliant Violet 605TM, anti-Ly6C-Brilliant Violet 421TM, anti-F4/80-APC, anti-CD8-Brilliant Violet 785TM, anti-granzyme B-Alexa Fluor^®^ 700, and anti-Foxp3-Alexa Fluor^®^ 647 (BioLegend, San Diego, CA, USA). The samples were washed with a staining buffer (2% FBS in PBS), resuspended in the staining buffer, and analyzed using the CytoFLEX 13-color cytometry (Beckman Coulter, Brea, CA, USA). The expression levels of immune cells were quantified using the CytExpert analysis software version 2.3.0.84 (Beckman Coulter, Brea, CA, USA).

### 2.12. Immunohistochemistry Staining

Tumor specimens were formalin-fixed, paraffin-embedded, and cut into 3 μm thick slides. Samples were deparaffinized with xylene for 15 min and sequentially rehydrated with different concentrations of ethanol. The samples were heat-inactivated in 10 mM sodium citrate buffer (0.05% Tween-20, pH 6.0) for 40 min at 100 °C for antigen retrieval, and then blocked using the BlockPRO blocking buffer (Visual Protein, Taipei, Taiwan) for 30 min at room temperature. Samples were then incubated with primary antibodies against STING (1:100; Cell Signaling Technology, Danvers, MA, USA) for 2 h at room temperature. To exclude non-specific signals, a negative control without the STING antibody was prepared for use. Endogenous peroxidase activity was inactivated by incubation with 3% hydrogen peroxide at room temperature for 10 min. The secondary antibody SignalStain^®^ Boost IHC Detection Reagent (HRP, Rabbit) (Cell Signaling Technology) was used and incubated for 30 min at room temperature. Subsequently, 3,3-diaminobenzidine (DAB) was used as a chromogen for 10 min using the DAB Substrate–Chromogen system (Dako Liquid DAB + Substrate Chromogen System; Agilent Dako, Santa Clara, CA, USA). The slides were washed with PBS and then counterstained with hematoxylin (Merck, Darmstadt, Germany), dehydrated with ethanol, stabilized with xylene, and mounted using a mounting solution (Merck). Cells expressing STING were visualized, and images were captured using a BX51 light microscope. Cells expressing STING were obtained from five different areas and scored as follows: 0, no expression; 1, mild expression; 2, moderate expression; and 3, severe expression, to indicate the different protein expression levels.

### 2.13. Statistical Analysis

The data are expressed as mean ± standard deviation, and one-way analysis of variance (ANOVA) with post hoc multiple comparison analysis was performed with an LSD test. Statistical analysis shows the significant differences between the control and ciprofloxacin-treated groups: * *p* < 0.05, ** *p* < 0.01, and *** *p* < 0.001. Significant differences between the ciprofloxacin and STING siRNA or STING siRNA plus ciprofloxacin-treated groups are indicated by ^#^ *p* < 0.05. Significant differences between ciprofloxacin plus anti-PD1 and anti-PD1 or ciprofloxacin-treated groups are indicated by ^#^ *p* < 0.05.

## 3. Results

### 3.1. Ciprofloxacin Induces dsDNA Expression

**Figure 1:**
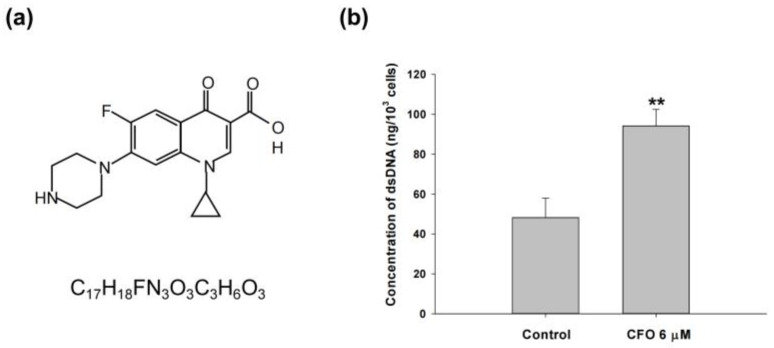
Double-stranded DNA (dsDNA) expression after ciprofloxacin treatment. The chemical formula and structure of the antibiotic ciprofloxacin (**a**). Mouse colorectal adenocarcinoma CT26 cells were managed with ciprofloxacin for 24 h, and the cytosolic dsDNA content was calculated by subtracting the amount of single-stranded DNA from that of the total DNA using spectrophotometer measurements (**b**). Data are presented as mean ± standard deviation and expressed relative to cells treated with vehicle alone. Statistically significant differences that compare the control with the ciprofloxacin-treated groups are indicated as ** *p* < 0.01.

We first evaluated the effect of ciprofloxacin on dsDNA production. Cytosolic dsDNA increased after treatment with 6 μM ciprofloxacin for 24 h (Figure 1b). This indicates that ciprofloxacin may activate the cGAS-STING signaling pathway.

### 3.2. Ciprofloxacin Does Not Affect Cell Viability, Morphology, or Cell Cycle in CT26 Colon Cells

**Figure 2:**
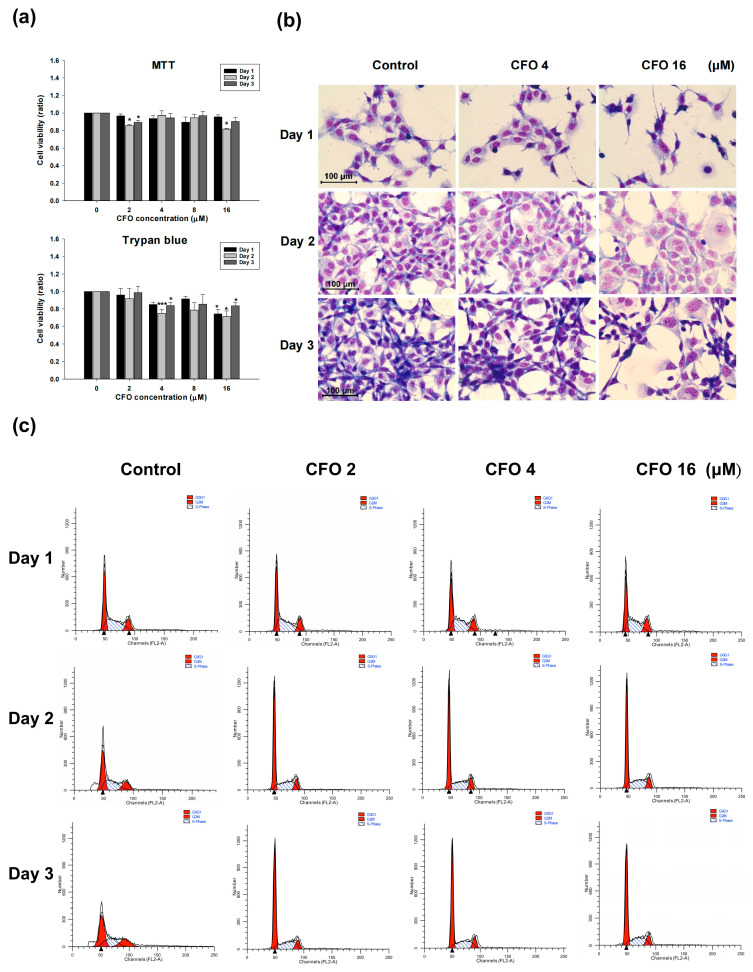
The cell viability, morphology, and cell cycle analysis of CT26 cells after ciprofloxacin treatment. Colorectal CT26 cells were challenged with diverse concentrations of ciprofloxacin for 24, 48, and 72 h, and cell viability was determined using the MTT and trypan blue exclusion assays (**a**). The morphology was analyzed with Liu’s staining (red color for cytoplasm and blue color for nuclei) and photographed (**b**). At the end of treatment, CT26 cells were collected and stained with propidium iodide, and flow cytometry was used to analyze cell cycle distribution (**c**). Data are presented as mean ± standard deviation and expressed relative to cells treated with the vehicle alone. Statistically significant differences that compare the control with the ciprofloxacin-treated groups are indicated as * *p* < 0.05 and *** *p* < 0.001.

The different concentrations of ciprofloxacin (0 to 16 μM) were added to CT26 colon cells for 24, 48, and 72 h, and cell viability and proliferation were estimated using the MTT reduction assay and trypan blue exclusion, respectively. The results demonstrated that ciprofloxacin had no impact on the viability of CT26 cells. However, CT26 cell proliferation was slightly inhibited at the highest concentration of ciprofloxacin (Figure 2a). The morphology of CT26 cells was visualized after ciprofloxacin treatment using Liu’s staining. The morphology of CT26 cells was not altered after treatment with low or high concentrations of ciprofloxacin for 24, 48, and 72 h (Figure 2b). The DNA histogram distribution analysis using flow cytometry revealed that ciprofloxacin did not affect the cell cycle of CT26 cells (Figure 2c). The percentages of cells in each phase of the cell cycle are shown in Table 1.

### 3.3. Ciprofloxacin Triggers Cytosolic DNA Formation and Activates cGAS-STING Signaling Pathway

**Figure 3:**
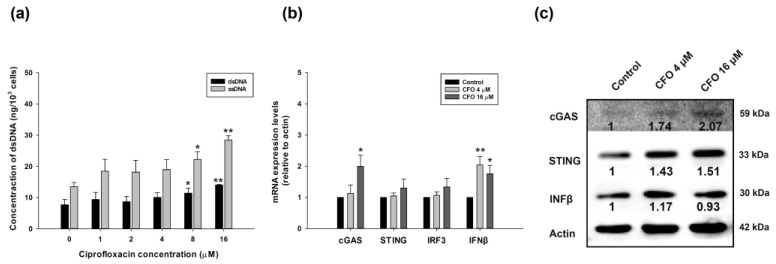
Double-stranded DNA (dsDNA), cyclic GMP-AMP synthase (cGAS)-stimulator of interferon gene (STING) pathway mRNA, and protein expression after ciprofloxacin administration in mouse colorectal adenocarcinoma CT26 cells. Ciprofloxacin was added to CT26 cells for 24 h. Cells were harvested, and a lysis buffer was used to disrupt the cells. The supernatant was collected to isolate cytosolic DNA using centrifugation. The dsDNA concentration was assessed by measuring the absorbance of wavelength at 260 nm (**a**). After treatment, total RNA was isolated and reverse transcribed to cDNA. The mRNA expression of cGAS, STING, interferon regulatory factor 3 (IRF3), and interferon-beta (IFNβ) was quantitated using gene-specific primers (**b**). For cGAS, STING, and IFNβ protein expression, cells were harvested and lysed, and the protein concentration after treatment was determined. Equal amounts of proteins were subjected to sodium dodecyl sulfate–polyacrylamide gel electrophoresis and transferred to polyvinylidene difluoride membranes, and the immunoblots were detected using chemiluminescent systems using a charge-coupled device image system. The blots are representative of cGAS, STING, and IFNβ proteins (**c**). Data are presented as mean ± standard deviation and expressed relative to cells treated with the control alone. Statistically significant differences that compare the control with the ciprofloxacin-treated groups are indicated as * *p* < 0.05 and ** *p* < 0.01.

The cytosolic DNA formation was further validated after ciprofloxacin treatment. Ciprofloxacin induced the formation of cytosolic dsDNA and ssDNA in a concentration-dependent manner after 24 h of treatment in CT26 adenocarcinoma cells (Figure 3a). The formation of dsDNA and ssDNA was significantly increased in treatments with higher concentrations of ciprofloxacin, i.e., 8 and 16 μM. In the cytosolic DNA-sensing cGAS-STING signaling pathway, IFNβ mRNA expression increased in ciprofloxacin treatment at a low concentration of 4 μM; however, the mRNA expression of cGAS, STING, and IRF3 did not change. The expression of cGAS and IFNβ mRNA was elevated after treatment with a high concentration of ciprofloxacin of 16 μM. STING and IRF3 mRNA levels increased but not significantly after treatment with a high concentration of ciprofloxacin (Figure 3b). The results imply that the activation of mRNA expression of cGSA-STING pathway at a high concentration of ciprofloxacin is needed. The expression of these proteins was also assessed, and the results showed that cGAS, STING, and IFNβ protein expression was elevated after ciprofloxacin treatment for 24 h (Figure 3c).

### 3.4. STING Small Interfering RNA (siRNA) Abrogates Ciprofloxacin-Induced Activation of cGAS-STING Pathway

**Figure 4:**
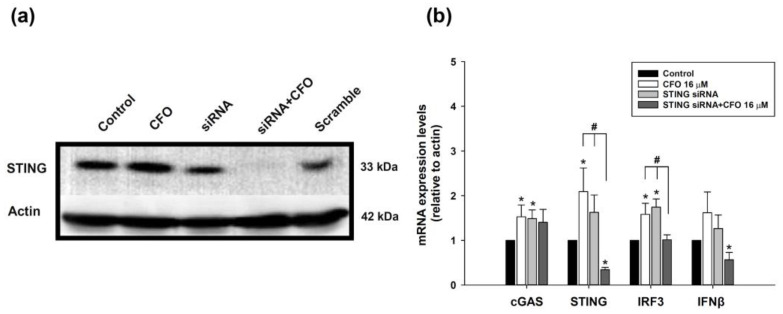
STING protein and cGAS, STING, IFR3, and IFNβ mRNA expression after ciprofloxacin, STING small interfering RNA (siRNA), or ciprofloxacin plus STING siRNA treatment. Mouse colorectal adenocarcinoma CT26 cells were treated with ciprofloxacin (CFO), STING siRNA, or ciprofloxacin plus STING siRNA for 24 h. The STING protein (**a**) and mRNA expression of molecules in the cGAS-STING signaling pathway were detected (**b**). Data are presented as mean ± standard deviation and expressed relative to cells treated with the vehicle alone. Statistically significant differences that compare the control with the treated groups are indicated as * *p* < 0.05. Statistically significant differences between the ciprofloxacin-treated and STING siRNA or STING siRNA plus ciprofloxacin-treated groups are indicated as ^#^ *p* < 0.05.

To further verify the involvement of ciprofloxacin in the activation of the cGAS-STING pathway, STING siRNA was administered to visualize STING protein expression in tumor cells. STING protein expression was induced by ciprofloxacin treatment, and STING siRNA moderately blocked STING expression. Co-treatment with STING siRNA and ciprofloxacin completely inhibited STING protein expression (Figure 4a). The mRNA expression levels of cGAS, STING, IRF3, and IFNβ increased after ciprofloxacin treatment. However, co-treatment with STING siRNA and ciprofloxacin decreased STING, IRF3, and IFNβ expression levels, but not that of cGAS (Figure 4b).

### 3.5. Ciprofloxacin Enhances Anti-PD1 Efficacy Against Tumor Burden Without Major Toxicity

**Figure 5:**
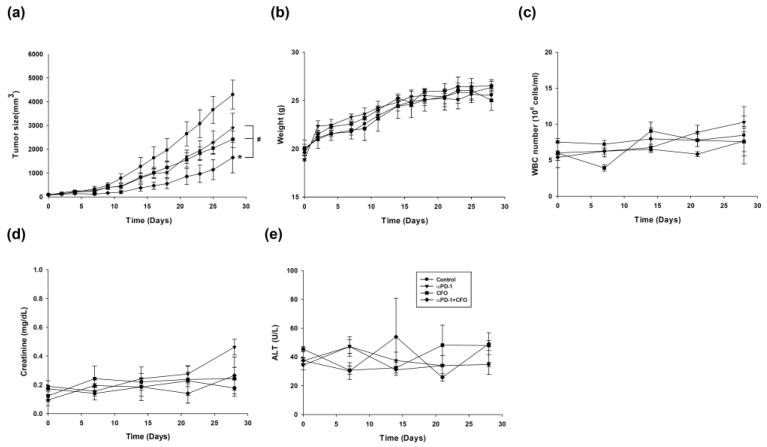
The curative effects and toxicities in the CT26 syngeneic animal tumor model after ciprofloxacin, anti-programmed cell death protein-1 (anti-PD1), and ciprofloxacin combined with anti-PD1 treatment. CT26 colorectal cells were implanted into the hind limb of BALB/c mice. After the tumor reached a size of 0.5 cm, the mice were administered with ciprofloxacin, anti-PD1 antibody, or ciprofloxacin combined with anti-PD1 antibody. The tumor size was recorded by a single observer and calculated (**a**). Toxicities were assessed by body weight (**b**), white blood cells (WBCs) for hematological parameter (**c**), creatinine for renal function (**d**), and alanine aminotransferase (ALT) for liver function (**e**). Data are presented as mean ± standard deviation and expressed relative to cells treated with the vehicle alone. Statistically significant differences between the control and combination-treated groups are indicated as * *p* < 0.05. Statistically significant differences between the single agent-treated and combination-treated groups are indicated as ^#^ *p* < 0.05.

The results of animal experiments showed that treatment with ciprofloxacin or anti-PD1 alone inhibited tumor growth, with a moderate effect on the CT26 subcutaneous tumor animal model. The growth inhibition due to ciprofloxacin and anti-PD1 alone was augmented by the combination of ciprofloxacin and anti-PD1 in the colorectal tumor model (*p* = 0.033 for anti-PD1 and *p* = 0.038 for ciprofloxacin, respectively) (Figure 5a). The toxicities of monotherapy and combination therapy were evaluated using body weight (Figure 5b), white blood cells (Figure 5c), creatinine to represent renal function (Figure 5d), and alanine transaminase to represent liver function (Figure 5e), and these biological parameters were not altered during treatment.

### 3.6. In Vivo Expression Profiles of Immune Cells in Tumor Microenvironment and Spleen

**Figure 6:**
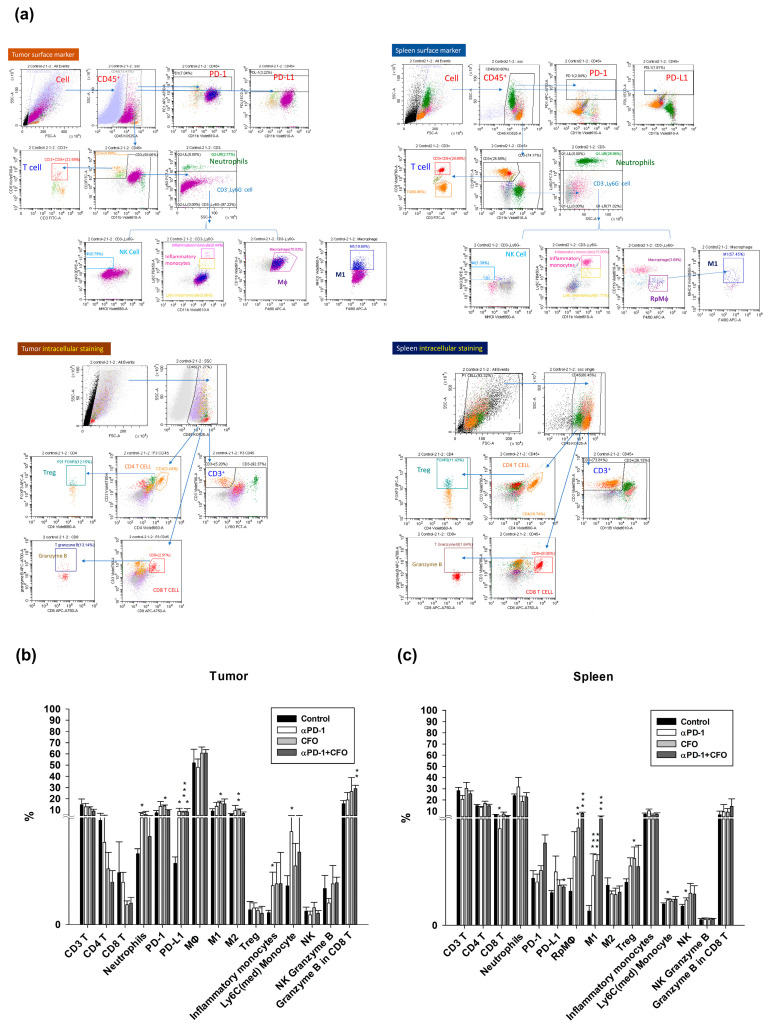
The distribution of various types of immune cells in the spleen and tumor microenvironment after a single agent, ciprofloxacin or anti-PD1 antibody, or the combination of ciprofloxacin and anti-PD1 antibody was administered in the CT26 syngeneic animal model. After four weeks, the mice were euthanatized, and the spleen and tumor samples were excised for the further analysis of immune cell profiles using flow cytometry. The gating strategies to isolate various kinds of immune cells using specific markers based on conjugated fluorescence are indicated (**a**). The expression percentage of distinct types of immune cells in the tumor (**b**) and spleen (**c**) specimens on day 28 after tumor implantation. Data from four mice of each group are expressed as mean ± standard deviation. Statistically significant differences between the control group and the ciprofloxacin-treated, anti-PD1-treated, or combination treatment groups are indicated by * *p* < 0.05, ** *p* < 0.01, and *** *p* < 0.001.

The expression profiles of immune cells in the TME and spleen were analyzed using flow cytometry. The gating strategy used to select the different subsets of immune cells for tumor and spleen samples is shown in Figure 6a. Data analysis revealed that, in the T cell populations, CD3, CD4, and CD8 T cells did not change after treatment in the tumor and spleen. Ciprofloxacin treatment augmented Treg expression in the spleen but not in the tumor. In the spleen, monotherapy with anti-PD1 and ciprofloxacin and combination therapy with anti-PD1 and ciprofloxacin induced M1 macrophage expression, but not M2 macrophage expression. Only ciprofloxacin treatment induced M1 and M2 macrophage expression in tumors. Ciprofloxacin monotherapy and combination therapy elicited red pulp macrophage (Rp Mφ) production in the spleen; however, the macrophage expression in the TME was not altered after the different treatments. Anti-PD1 treatment generated inflammatory and Ly6C(med) monocyte expression in the tumor; however, it only increased Ly6C(med) monocyte expression in the spleen. Anti-PD1 treatment also induced the expression of NK cells in the splenic microenvironment. Anti-PD1 and ciprofloxacin co-treatment induced CD8^+^ T cells granzyme B expression in the tumor but not in the spleen (Figure 6b,c).

### 3.7. STING Protein Expression in Tumor Specimens

**Figure 7:**
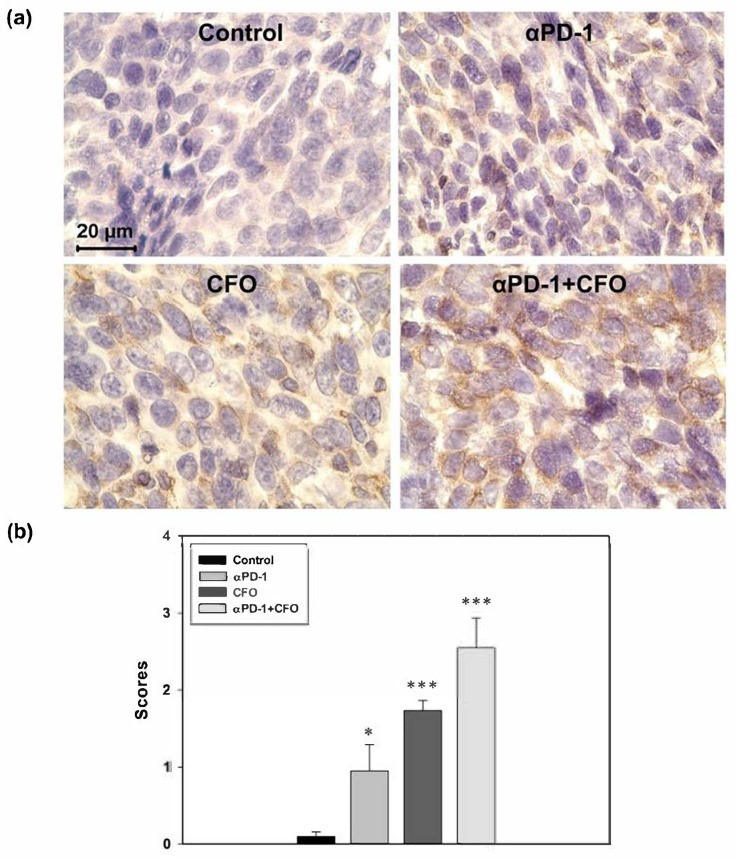
The STING protein expression (brown color) levels in the tumor after the ciprofloxacin, anti-PD1, or combination treatment of the CT26 syngeneic animal model. The tumor specimens were excised on day 28 and immunohistochemically stained with the STING protein antibody and hematoxylin (blue color) (**a**). The expression levels of STING protein in the tumor after treatment were recorded, calculated, and scored (**b**). Data are presented as mean ± standard deviation and expressed relative to mice treated with the vehicle alone. Statistically significant differences between the control group and the monotherapy-treated or combination-treated groups are indicated as * *p* < 0.05 and *** *p* < 0.001.

The immunohistochemical staining of tumor specimens revealed that STING protein expression increased after monotherapy and combination therapy. STING protein expression after ciprofloxacin treatment was higher than that after anti-PD1 treatment. The STING expression level after combination therapy was higher than that after anti-PD1 or ciprofloxacin monotherapy (Figure 7).

### 3.8. M1 Macrophage Is Not Associated with cGAS-STING Pathway Activation

**Figure 8:**
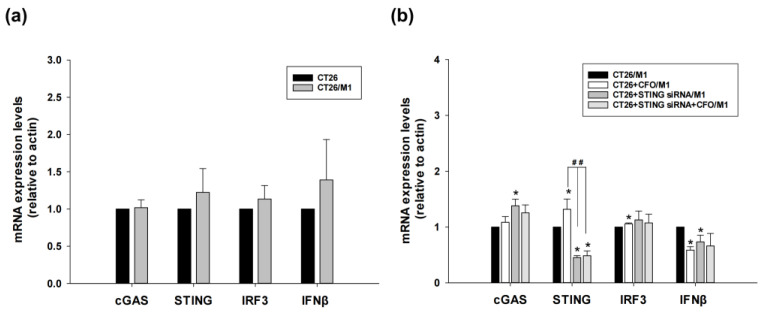
The mRNA expression of proteins in the cGAS-STING pathway signaling pathway after STING siRNA or ciprofloxacin treatment in the CT26 and mouse M1 macrophage co-culture system. Mononuclear cell layers were isolated from the mice spleen, and M1 macrophages were induced by exposure to lipopolysaccharide and interferon-γ. The M1 macrophages were co-cultured with CT26 cells, and the mRNA expression of cGAS, STING, IRF3, and IFNβ in CT26 cells was quantitated using gene-specific primers (**a**). Treatment with ciprofloxacin (CFO), siRNA STING, and ciprofloxacin plus siRNA STING in the CT26/M1 macrophage co-culture system. The mRNA expression of target genes was quantitated using gene-specific primers (**b**). Data are presented as mean ± standard deviation and expressed relative to cells treated with CT26 or CT26/M1 macrophage alone. Significant differences between the control and treatment groups are indicated as * *p* < 0.05. Significant differences between the ciprofloxacin-treated group and the STING siRNA or STING siRNA plus ciprofloxacin-treated groups in the co-culture system are indicated as ^##^ *p* < 0.01.

The role of M1 macrophages was also evaluated. cGAS, STING, IRF3, and IFNβ mRNA expression levels in CT26 cells were not changed after mouse M1 macrophages isolated from the spleen were co-cultured with CT26 cells compared with CT26 cells only (Figure 8a). Ciprofloxacin induced STING and IRF3 mRNA expression in the co-culture system. Pretreatment with STING siRNA inhibited STING and IFNβ expression in co-cultured CT26 cells and M1 macrophages, as well as after ciprofloxacin treatment (Figure 8b).

## 4. Discussion

In this study, we validated the ciprofloxacin-induced cGAS-STING signaling pathway activation in CRC. The activation of this DNA-sensing pathway may be associated with enhanced anti-PD1 activity in colon cancer treatment.

The putative mechanism of action of ciprofloxacin involves the disruption of bacterial DNA topoisomerase II and gyrase activity to inhibit DNA replication. This function allows ciprofloxacin to treat Gram-positive and -negative bacterial infections [17]. However, we found a novel effect of ciprofloxacin in promoting cytosolic DNA formation and the activation of the cGAS-STING signaling pathway. This novel effect may account for the enhanced anti-PD1 effect. Previous studies have reported that ciprofloxacin also inhibits anti-tumor growth in several cancer cell lines, including colorectal carcinoma cells, triple-negative breast cancer, bladder cancer, and prostate cancer [21,22,23,24,25]. Ciprofloxacin suppresses DNA synthesis; disrupts mitochondrial membrane potential; upregulates Bax and caspase-3, -8, and -9; and enhances apoptosis in rat and human CRC cells [21]. Ciprofloxacin induces cell cycle arrest at the S/G2-M phase and the expression of cell cycle-related proteins TP53 and CDKN1 and pro-apoptotic proteins in bladder and prostate cancers [22,23,24]. Similar mechanisms have been demonstrated to elevate p53 expression and trigger apoptosis via the activation of the p53/Bax/Bcl-2 signaling pathway in MDA-MB-231 breast cancer cells after ciprofloxacin treatment [25]. In our study, we found that cell viability, morphology, and cell cycle were not altered by ciprofloxacin; however, cell proliferation was only slightly inhibited. This discrepancy may be due to the different cell lines and ciprofloxacin concentrations used in the experiments. The concentration of ciprofloxacin in these studies ranged from 50 to 500 μg/mL (approximately 150–1500 μM), which was much higher than that used in our study. Another issue is that ciprofloxacin does not impact the cell cycle but induces cytosolic DNA elevation. This may be the putative mechanism of ciprofloxacin shown to inhibit topoisomerase II activity and cause double-stranded DNA break and DNA damage. The use of higher concentrations of ciprofloxacin to verify tumor growth inhibition, cell cycle arrest, and apoptosis in mouse colorectal CT26 cells should be further investigated.

Antimicrobial fluoroquinolones are widely used in clinical practice. Fluoroquinolones inhibit enzymatic activity to prevent DNA ligation, resulting in single- and double-stranded DNA breaks [26]. Ciprofloxacin inhibits bacterial DNA gyrase and topoisomerase IV, leading to double-stranded break formation. dsDNA is further processed into single-stranded DNA for binding to the RecA protein and for DNA repair. In mammalian cells, ciprofloxacin interacts with topoisomerase II, leading to double-stranded breaks and ultimately cell death. Some ciprofloxacin derivatives have been reported that target topoisomerase I/II to form single-stranded and double-stranded breaks, thereby inducing tumor cell apoptosis [27]. The mechanisms of ciprofloxacin increase the amount of cytosolic double-stranded DNA (dsDNA) and single-stranded DNA (ssDNA). In human cells, two isozymes of topoisomerase II, IIα and IIβ, are encoded and involved in cancer development [28]. Therefore, exploring fluoroquinolones as anticancer drugs that target human topoisomerase II is a reasonable option. Hangas et al. reported that ciprofloxacin inhibited mitochondrial topoisomerase II activity, followed by the silencing of mitochondrial transcription, replication, and aberrant mitochondria, resulting in the disturbed proliferation and differentiation of C2C12 cells [29]. Ciprofloxacin causes a reduction in mitochondrial DNA (mtDNA) content, energy formation, and calcium homeostasis in leukemia Jurkat cells [30]. The cytotoxicity of ciprofloxacin is derived from site-specific, double-stranded mtDNA breaks and the loss of mtDNA in tumor cells [31]. These observations indicate that ciprofloxacin can target mitochondrial DNA and release dsDNA in the cytosol. Another study demonstrated that using plasmid DNA and treating with high concentrations of ciprofloxacin (200–300 μM) inhibited human topoisomerase IIα and IIβ activity and led to DNA breaks but not at low concentrations (3–30 μM [approximately 1–10 μg/mL]), which is expected to be similar to the serum ciprofloxacin levels in patients [32]. Ciprofloxacin specifically targets bacterial topoisomerases but does not have the same effect on human topoisomerases, which is crucial for its antibacterial activity while minimizing harm to human cells [32]. Mitochondrial damage by cisplatin induces mitochondrial DNA release into the cytosol, with the subsequent activation of the cGAS-STING signaling pathway [33]. In our study, ciprofloxacin induced ssDNA and dsDNA formation in the cytosol and elevated the levels of component molecules of the cGAS-STING pathway. However, whether ssDNA and dsDNA originate from mitochondrial or chromatin DNA remains unknown. Mitochondria originated from endosymbiotic Alphaproteobacteria and integrated into eukaryotic cells [34]. We speculate that ciprofloxacin may cause mitochondrial DNA breaks in CT26 cells by inhibiting topoisomerase II activity by subsequently forming ssDNA and dsDNA, and releasing them into the cytosol, thus further activating the cGAS-STING pathway. This suggests that mitochondrial DNA could act as a damage-associated molecular pattern signal for cGAS recognition and cause an innate immune system response [35]. However, we cannot rule out the possibility that ciprofloxacin damages the nucleus and releases DNA into the cytosol after treatment with high concentrations of ciprofloxacin.

Topoisomerases relax supercoiled DNA and regulate its topological structure. Topoisomerases are responsible for DNA replication, transcription, genome integrity, and regulation of cell cycle progression. Topoisomerases have been used as targets for the design and development of anticancer drugs that disrupt their activity. Topoisomerase II inhibitors are subdivided into two categories: Topo II catalytic inhibitors and Topo II poisons [36]. Ciprofloxacin is a Topo II poison inhibitor that increases the covalent complexes of DNA and topoisomerase II, forming double-stranded breaks, and is toxic to bacteria. Other Topo II poisons that have been developed as clinical chemotherapy drugs include doxorubicin, etoposide, and teniposide [36]. Teniposide has been reported to induce the expression of the MC38 and CT26 tumor cell immunogenic cell death marker HMGB1, T cell activation, and DC maturation. Teniposide induces tumor immunogenicity by activating NF-κB and STING-dependent type I IFN production [19]. Ultrasound-responsive low-dose doxorubicin liposomes enhance doxorubicin release into the tumor cell nucleus and induce tumor mitochondrial oxidation. The released tumor mitochondria DNA triggers the cGAS-STING pathway activation and enhances T cell immunity [37]. Another report demonstrated that ciprofloxacin causes nuclear and mitochondrial DNA release into the cytosol, mitochondrial damage, the formation of reactive oxygen species, and STING protein activation in the mouse aorta [38]. These results imply that topoisomerase II inhibitors, including ciprofloxacin, can cause harmful nuclear or mitochondrial DNA release into the cytosol for DNA-sensing signaling and cGAS-STING pathway activation.

However, the safety of using ciprofloxacin is a concern. The most common adverse effects of fluoroquinolones, including ciprofloxacin, are gastrointestinal disorders. Other severe side effects include tendinopathy, peripheral neuropathy, QTc prolongation, cardiac arrhythmias, neuropsychiatric disorders, aortic aneurysms, and aortic dissection [17,39]. Clinically relevant studies have shown that adverse effects are mild after oral administration. In our in vivo study, ciprofloxacin did not show any biological toxicity, including effects on body weight, liver and renal function, or hematological profiles. The results imply that, in our system, low-dose ciprofloxacin is harmless compared to orally administered ciprofloxacin twice a day for 1–2 weeks at doses of 250–750 mg, according to the severity of infections in the clinic. Furthermore, a previous report showed that patients with cancer who were orally administered 750 mg of ciprofloxacin every 8 h experienced mild side effects, mostly gastrointestinal disorders [40]. Ciprofloxacin is effective and safe for treating infections in patients with cancer [40]. However, toxicities and side effects should be monitored after a clinical dose of ciprofloxacin is administered in in vivo anti-tumor studies in the future.

The topoisomerase II inhibitor, teniposide, has been reported to enhance anti-PD1 anti-tumor efficacy in mouse CRC. Teniposide-potentiated immune checkpoint blocker anti-tumor immunity is associated with intratumor STING activation [19]. These results are consistent with our results showing that ciprofloxacin potentiates the anti-PD1 anti-tumor effect in a CT26 syngeneic animal model. Tumor specimens also showed the highest expression of STING protein with the combination therapy. Ciprofloxacin exerts a synergistic effect on tumor growth suppression in combination with immunotherapy. Another study showed that the topoisomerase II inhibitor idarubicin could sensitize anti-PD1 immunotherapy by CD8^+^ T cells and activate the TME immune status in hepatocellular carcinoma [41]. However, a retrospective cohort study showed that concurrent treatment with antibiotics and immune checkpoint inhibitors was associated with higher mortality in patients with hepatocellular carcinoma [42]. Broad-spectrum antibiotics have demonstrated negative outcomes with immune checkpoint inhibitor treatment in patients with cancer in clinical settings. These results shed light on how antibiotics modulate the gut microbiota and induce dysbiosis to influence immunotherapy efficacy [43]. The gut microbiome plays an important role in immune function and is a potential target of immunotherapy. Antibiotics disrupt the microbiota balance and hinder the efficacy of immunotherapy. Retrospect studies demonstrated that antibiotic administration is inconsistent. However, whether antibiotics are beneficial for immunotherapy remains unclear. The results remain controversial because the dosage, duration, and types of antibiotic treatment should be considered when patients with cancer receive immunotherapy. The favorable outcomes of the combination of ciprofloxacin and immunotherapy should be investigated in more detail. More research is needed to understand the specific mechanisms by which ciprofloxacin modulates the immunotherapy response and develop a new regimen for patients to enhance immunotherapy efficacy after ciprofloxacin use. Next-generation sequencing technology such as 16S rRNA sequencing and metagenomics, dietary interventions, microbiota depletion models, or fecal microbiota transplantation methods could be used to analyze or modulate the gut microbiota and to verify the mechanisms of concurrent ciprofloxacin and immunotherapy treatment that influence the gut microbiota in the future.

Many studies have shown that ciprofloxacin exerts chemosensitizing effects. The combination of ciprofloxacin with chemotherapeutic drugs such as doxorubicin and docetaxel enhance hormone-refractory prostate cancer cell growth inhibition [44]. Ciprofloxacin reverses multidrug resistance and enhances tumor cell-sensitized ABCB1 substrates to maintain chemo-drug concentration in cells [45]. Geller et al. found that ciprofloxacin abrogates the bacteria-induced conversion of the chemo-drug gemcitabine into its inactive form to resist tumor effects [46]. This indicates that ciprofloxacin has the potential to be developed as a combination therapy with other clinical chemotherapeutic drugs to improve cancer treatment.

## 5. Conclusions

In conclusion, the inexpensive clinical drug ciprofloxacin, a fluoroquinolone-class antibiotic, may activate the cGAS-STING signaling pathway and enhance the anti-PD1 therapeutic effects in colorectal cancer. Immune cell profiles in the TME were also modified by ciprofloxacin.

## Figures and Tables

**Table 1:** The percentage of CT26 cells in the G0/G1, S, and G2/M phases of the cell cycle after treatment with ciprofloxacin for 1, 2, and 3 d.

		G0/G1	S	G2/M
Day 1	Control	41.6 ± 0.0	43.3 ± 2.3	15.1 ± 2.3
	CFO 2 μM	39.4 ± 1.5	42.5 ± 1.2	18.1 ± 2.0
	CFO 4 μM	36.9 ± 1.1	46.2 ± 2.4	16.9 ± 2.2
	CFO 16 μM	37.2 ± 0.4	46.6 ± 1.9	16.2 ± 2.3
Day 2	Control	45.1 ± 1.8	41.2 ± 1.0	13.8 ± 0.9
	CFO 2 μM	49.6 ± 6.7	33.7 ± 6.6	16.7 ± 3.7
	CFO 4 μM	53.6 ± 2.9	31.2 ± 5.2	15.1 ± 3.1
	CFO 16 μM	50.9 ± 3.6	37.0 ± 2.2	12.1 ± 1.5
Day 3	Control	54.6 ± 8.5	33.4 ± 4.7	12.1 ± 3.8
	CFO 2 μM	60.3 ± 0.9	28.8 ± 1.6	10.9 ± 2.0
	CFO 4 μM	59.1 ± 2.7	29.4 ± 0.5	11.5 ± 2.2
	CFO 16 μM	61.3 ± 2.9	26.3 ± 5.0	12.4 ± 2.1

## Data Availability

The datasets presented in this study are included in the article. further inquiries can be directed to the corresponding author.
